# The feasibility of one-stage flexible ureteroscopy lithotripsy in solitary kidney patients with 1–3 cm renal stones and risk factors of renal function changes

**DOI:** 10.1080/0886022X.2021.1872625

**Published:** 2021-01-24

**Authors:** Yang Pan, Han Chen, Hualin Chen, Xiaoxiang Jin, Yunxiao Zhu, Gang Chen

**Affiliations:** Department of Urology, The First Affiliated Hospital of Chongqing Medical University, Chongqing, China

**Keywords:** Flexible ureteroscopy lithotripsy, solitary kidney, renal stones, prior stenting, ureteral stent, glomerular filtration rate

## Abstract

**Purpose:**

To compare perioperative outcomes and long-term renal function changes between prior stenting (PS) and not prior stenting (NPS) before flexible ureteroscopy lithotripsy (f-URS) for solitary kidney patients.

**Methods:**

Solitary kidney patients with 10–30 mm renal stones were enrolled in this historical control study. Perioperative parameters and complications were compared. Stone-free was defined as the absence of any residual stones on a CT scan. Renal function changes were evaluated by estimated glomerular filtration rate (eGFR) and adjusted for body surface area. A decrease in the eGFR over 20% was identified as ‘deterioration’ in renal function. The follow-up period was at least 6 months. Logistic regression was used to identify risk factors of renal function deterioration.

**Results:**

Of the 76 patients included, 40 cases experienced prior stenting before f-URS. The average stone diameter was 16.8 ± 4.7 mm, ranging from 10.0 to 28.4 mm. Initial SFR was 85.0 and 83.3% in the PS and NPS groups, respectively (*p* = 0.842), while SFR after the second procedure was 97.5 and 94.4% (*p* = 0.926). Seven PS and 5 NPS patients developed complications (*p* = 0.666). At the postoperative 6 months, seven patients showed a deteriorated renal function. Surgical time in minutes was identified as a risk factor for renal function deterioration after the operation (OR = 1.061, 95% CI: 1.015–1.109, *p* = 0.009, per minute).

**Conclusion:**

It appears that one-stage f-URS without PS could be feasible for 10–30 mm renal stones in solitary kidney patients, and less surgical time might be beneficial to protect renal function.

## Introduction

The solitary kidney is becoming more and more common due to the reasons such as contralateral nephrectomy and nonfunctional kidney. Renal stones in patients with a solitary kidney are also not a rare urological disease. The treatment for these patients is challenging and significant because any damage to a solitary kidney may result in severe comorbidities. Therefore, endoscopic procedures with no wound for renal stones are preferred by patients with a solitary kidney [1]. Many previous studies have reported the safety and efficacy of flexible ureteroscopy lithotripsy (f-URS) for renal stones in solitary kidney patients and recommended f-URS as the first choice [2–6].

Arguments exist over many years about whether prior stenting for 2 weeks before f-URS. Some urologists consider that prior stenting before f-URS can increase the surgical success rate and stone-free rate (SFR) [7], while others regard prior stenting as unnecessary for bilateral kidney stones except in some special patients [8]. Similarly, it is hard to make a definite choice between one-stage f-URS and prior stenting firstly for renal stones in patients with a solitary kidney. To date, there are few reports on whether prior stenting is necessary when choosing f-URS for renal stones in solitary kidney patients. The present study aims to compare perioperative outcomes and long-term renal function changes of f-URS in solitary kidney patients with 10–30 mm renal stones with and without prior stenting.

## Patients and methods

### Patients

Solitary kidney patients with 10–30 mm renal stones managed by f-URS between June 2015 to June 2019 were retrieved from electronic medical records of the First Affiliated Hospital of Chongqing Medical University. All f-URS procedures included in the present study were performed by the same experienced surgeon. Before November 2017, all patients with a solitary kidney and renal stones were required to indwell a prior stent before f-URS in our department. Since November 2017, the surgeon decided to perform one-stage f-URS without prior stenting for renal stones in solitary kidney patients, provided that patients did not experience emergency events or severe comorbidities such as ureteral obstruction, severe infections, and acute kidney injury. As a result, patients who performed f-URS with prior stenting were identified as the prior stenting (PS) group, and patients who performed one-stage f-URS without prior stenting were identified as the not prior stenting (NPS) group. A historical control study was conducted and two groups were compared along the lines of a historical switch of practice. The inclusion criteria consisted of age between 18 and 80 years and a history of f-URS for 10–30 mm renal stones in patients with a solitary kidney. Exclusion criteria were as follows: transplant solitary kidney, ureteral stones, staghorn stones, incomplete medical history records, untreated urinary infection, bleeding disease, severe urinary tract stricture, pregnancy, severe hydronephrosis, and acute renal insufficiency. Approval was obtained from the Institutional Ethics Committee of the First Affiliated Hospital of Chongqing Medical University (Human Research Committee Approval number: 2018-003).

### Clinical data

The etiology of solitary kidney in the present study included congenital renal agenesis, previous contralateral nephrectomy, and contralateral nonfunctional kidney. The contralateral nonfunctional kidney was defined as that patient’s contralateral renal function was less than 5% in a split renal function on a 99 m Tc labeled dimercaptosuccinic acid single-photon kidney emission computerized tomography (ECT), or computerized tomography (CT) scan showed contralateral kidney was significantly atrophic and intravenous pyelography (IVP) showed no urine secretion [9]. All the patients with positive urine culture were treated using drug-sensitive antibiotics preoperatively and f-URS was performed only when their urine culture results became negative.

Demographic characteristics, stone characteristics, short-term and long-term renal function changes, perioperative outcomes, and complications were assessed and compared in detail between the two groups. The data of the first f-URS procedure were analyzed mainly if the patients experienced multiple procedures. The surgical time in two groups began to be calculated when the guidewire was inserted from the ipsilateral ureterovesical opening. Therefore, the surgical time in the PS group did not include the time of the pre-stenting procedure and the time of removing the stent before f-URS.

The serum creatinine was measured using enzymatic assays (Roche Diagnostic, USA), calibrated with a traceable high-level isotope dilution mass spectrometry (IDMS) reference. It was a validated measurement of serum creatinine throughout the study period and there was not a technological switch taking place. The estimated glomerular filtration rate (eGFR) was calculated using the Chronic Kidney Disease Epidemiology Collaboration (CKD-EPI) equations based on age, sex, race, and serum creatinine levels (Supplementary file 1). Furthermore, each patient’s actual eGFR was adjusted for their body surface area (BSA). The actual eGFR was calculated by multiplying the value from CKD-EPI equations with their actual BSA and dividing 1.73 m^2^. Renal function changes were assessed by the actual eGFR levels.

Changes in eGFR levels before and after the procedures were also given as ‘percentage of change’, which was calculated as follows: ‘(the difference between timely level and baseline level)/baseline level × 100%’. An increase in the eGFR over 20% was considered as ‘improvement’, a decrease over 20% as ‘deterioration’, and changes within 20% as ‘stabilization’ in renal function. All complications were categorized using the Clavien–Dindo grading system [10].

### Prior stenting and f-URS procedures

Prior stenting was conducted using a routine method. Under local anesthesia, a guidewire was inserted from the ipsilateral ureterovesical opening to the renal pelvis using ureteroscopy. A 6-Fr double J stent was placed along the guidewire. After this procedure, an abdominal plain X-ray of the kidney, ureter, and bladder (KUB) was performed to confirm the stent was in the correct position. The indwelling time of the stent was approximately 2 weeks before f-URS.

Flexible ureteroscopy lithotripsy for renal stones in solitary kidney patients was performed under general anesthesia and standard lithotomy position. For patients with prior stenting, the stent was removed using forceps through ureteroscopy firstly. The subsequent procedures were the same for patients in two groups. A guidewire was inserted from the ipsilateral ureterovesical opening. Along the guidewire, the ureter was checked and expanded using ureteroscopy retrograde until reaching the renal pelvis. After ureteral stenosis or ureteral stone was excluded, a ureteral access sheath (UAS) with an inner/outer diameter of 12/14-Fr (Cook Urological, Bloomington, IN, USA) was placed into the ureter. A flexible ureteroscope was put into the renal pelvis and stones were searched during the pelvicalyceal system. After finding the stones, a 200-µm holmium laser at a power of 0.8–1.5 J and a pulse frequency of 10–20 Hz was used to crush the stones into fragments of less than 2 mm. The large fragments were removed by a nitinol stone basket under the vision of flexible ureteroscopy. At last, the pelvicalyceal system was examined again to ensure no large remaining stones. The post-ureteroscopic lesion scale (PULS) grading system [11] was used to assess the degree of ureteral lesions after withdrawing the UAS. If there were no severe complications such as fever and urinary sepsis, patients were discharged 1 day postoperatively.

If a standard size UAS (12/14-Fr) could not be inserted due to ureteral stenosis or distortion, a smaller size UAS with an inner/outer diameter of 10/12-Fr (Cook Urological, Bloomington, IN, USA) was used. If the smaller size UAS could not be inserted for the first time, ureteric balloon dilation under the vision of ureteroscopy was performed intraoperatively. Then, the smaller size UAS was attempted for the second time. If it still could not be inserted, two subsequent ways were provided based on patients’ or their family’s selection. One was to perform f-URS without UAS, the other was to insert a stent and to wait at least for two weeks.

### Stone-free rate

Stone-free was defined as the absence of any residual stones in the kidney or ureter. If a patient did not achieve the stone-free status, extracorporeal shock-wave lithotripsy (SWL) or second f-URS was conducted based on the size of residual stones and patients’ preference. Patients were assessed by a non-enhanced CT scan 1 month after each procedure to confirm the stone-free rate (SFR).

### Statistical analysis

Statistical analysis was performed with IBM Statistical Package for the Social Sciences (SPSS), version 23.0 for Windows. The independent samples student’s *t*-test was used for comparison of two groups when data showed normal distribution; otherwise, the Mann–Whitney *U* test was used. Pearson’s Chi-square test or Fisher’s exact test was used for categorical variables. Univariate and multivariate logistic regression was used to identify independent factors of renal function deterioration at postoperative 6 months after f-URS. Statistical significance was set as two-tailed *p* < 0.05.

## Results

### Patients’ demographics and clinical characteristics

In total, 76 solitary kidney patients with 10–30 mm renal stones, who were managed by f-URS, were included in our study. There were 40 and 36 patients in the PS and NPS group, respectively. The age ranged from 29 to 80 years, and 56.6% of all patients were male. The mean body mass index (BMI) was 24.3 ± 3.7 kg/m^2^. The greatest stone diameter was ranging from 10.0 to 28.4 mm and the average value was 16.8 ± 4.7 mm. The left and right side of renal stones were found in 36 cases and 40 cases, respectively. Twenty-six patients had previous contralateral nephrectomy history due to renal cell carcinoma (*n* = 15), renal pelvis carcinoma (*n* = 3), ureteral urothelial carcinoma (*n* = 4), severe purulent kidney (*n* = 1), tuberculosis of kidney (*n* = 2), and renal rupture hemorrhage (*n* = 1). Four cases had congenital renal agenesis and 46 patients were diagnosed with contralateral nonfunctional kidney.

Patients’ demographics and clinical characteristics (including age, sex, height, weight, BMI, solitary kidney etiology, preoperative comorbidities, urine culture results, and grading of patients for surgical procedures according to American society of anesthesiologists), and stone-related characteristics (including greatest diameter, number, location, and side) were shown in Table 1. These results were comparable between the two groups, with no statistically significant difference (Table 1).

**Table 1.** Demographic, clinical, and stone-related data.

	Total	PS	NPS	*p*-Value
Number of patients, *n*	76	40	36	
Age, years, mean ± SD	55.0 ± 12.3	55.0 ± 11.8	55.1 ± 13.0	0.947
Gender, *n* (%)				
Male	43 (56.6)	23 (57.5)	20 (55.6)	0.864
Female	33 (43.4)	17 (42.5)	16 (44.4)	
Weight, kg, mean ± SD	63.5 ± 11.6	63.7 ± 10.7	63.4 ± 12.7	0.907
Height, cm, mean ± SD	161.7 ± 8.8	160.7 ± 9.5	162.5 ± 8.0	0.449
BMI, kg/m^2^, mean ± SD	24.3 ± 3.7	24.6 ± 3.5	23.9 ± 4.0	0.406
Etiology of solitary kidney, *n* (%)				
Congenital renal agenesis	4 (5.3)	3 (7.5)	1 (2.8)	0.610
Contralateral nephrectomy	26 (34.2)	14 (35)	12 (33.4)	
Contralateral nonfunctioning kidney	46 (60.5)	23 (57.5)	23 (63.8)	
Preoperative comorbidities, *n* (%)				
Cardiovascular disease	23 (30.3)	9 (22.5)	14 (38.9)	0.120
Diabetes mellitus	6 (7.9)	4 (10)	2 (5.6)	0.677
Preoperative urine culture results, *n* (%)				
Positive	14 (18.4)	5 (12.5)	9 (250	0.160
Negative	62 (81.6)	35 (87.5)	27 (75)	
Preoperative ASA Grade, *n* (%)				
Grade 2	56 (73.7)	30 (75)	26 (72.2)	0.784
Grade 3	20 (26.3)	10 (25)	10 (27.8)	
Greatest stone diameter, mm, mean ± SD	16.8 ± 4.7	17.7 ± 4.8	15.7 ± 4.4	0.070
Greatest stone diameter >20 mm, mean ± SD	24.0 ± 2.2	24.6 ± 1.9	23.1 ± 2.4	0.156
Greatest stone diameter <20 mm, mean ± SD	14.7 ± 2.8	15.4 ± 2.9	14.0 ± 2.6	0.490
Stone number, *n*, mean ± SD	1.6 ± 0.9	1.5 ± 0.8	1.7 ± 1.0	0.490
Single stone, *n* (%)	47 (61.8)	26 (65)	21 (58.3)	0.550
Multiple stones, *n* (%)	29 (38.2)	14 (35)	15 (41.7)	
Greatest stone diameter, *n* (%)				
More than or equal to 20 mm	17 (22.4)	10 (25)	7 (19.4)	0.562
Less than 20 mm	59 (77.6)	30 (75)	29 (80.6)	
Stone side, *n* (%)				
Left renal stones	36 (47.4)	19 (47.5)	17 (47.2)	0.981
Right renal stones	40 (52.6)	21 (52.5)	19 (52.8)	
Stone location (main), *n* (%)				
Renal pelvis	29 (38.1)	15 (37.5)	14 (38.9)	0.577
Upper calix	5 (6.6)	4 (10)	1 (2.8)	
Middle calix	5 (6.6)	3 (7.5)	2 (5.5)	
Lower calix	37 (48.7)	18 (45)	19 (52.8)	

PS: prior stenting; NPS: not prior stenting; SD: standard deviation; BMI: body mass index; ASA Grade: Grading of patients for surgical procedures according to American Society of Anesthesiologists.

### Perioperative outcomes

The mean surgical time in total was 49.9 ± 22.3 min, which was 48.6 ± 22.6 and 51.4 ± 22.1 min in the PS and NPS groups, respectively; the difference of surgical time between the two groups was not statistically significant (*p* = 0.570). The average postoperative hospital duration was 2.2 ± 1.6 days (2.3 ± 1.5 vs. 2.1 ± 1.6 days, *p* = 0.186). The UAS was placed successfully for all patients (a small size UAS for 2 cases) in the PS group. However, the small size UAS still could not be inserted for 2 patients in the NPS group due to ureteral stenosis. According to their family’s request, f-URS without using UAS was attempted and performed successfully in these 2 special patients. As a result, the UAS placement success rate was 100% and 94.4% in the PS and NPS groups, respectively, without a statistical difference (*p* = 0.221). Except for 2 patients who experienced unsuccessful UAS insertion in the NPS group, everyone else used a nitinol stone basket smoothly. No patients occurred ureter injuries according to the results of the PULS grading system and follow-up of at least 6 months.

### Stone-free rate

The initial SFR after the first f-URS procedure was 85.0% and 83.3% in the PS and NPS groups, respectively (*p* = 0.842). Among 12 patients who did not achieve the stone-free status after the first f-URS procedure, SWL and second f-URS were performed for 4 and 8 cases, respectively. After the second procedure of f-URS or SWL, the SFR increased to 97.5% and 94.4% in the PS and NPS groups, respectively (*p* = 0.926). Three patients (1 in the PS group and 2 in the NPS group) had multiple stones and the greatest stone diameter was more than 25 mm. Eventually, they achieved the stone-free status after the third procedure. The main biochemical compositions of stones were analyzed postoperatively and showed no statistical difference between the two groups; calcium oxalate phosphate calculi (47.4%) and calcium oxalate monohydrate calculi (31.6%) were relatively more common (Table 2).

**Table 2.** Renal function changes and perioperative outcomes.

	Total (*n* = 76)	PS (*n* = 40)	NPS (*n* = 36)	*p*-Value
eGFR (adjusted for BSA) on, mL/min/1.73m^2^, mean ± SD				
Preoperatively	57.05 ± 19.37	57.34 ± 19.09	56.73 ± 19.94	0.891
Postoperative 1 month	57.52 ± 19.10	57.65 ± 20.27	57.38 ± 18.01	0.952
Postoperative 6 months	60.63 ± 20.54	60.45 ± 22.48	60.83 ± 18.46	0.937
Δ% eGFR at postoperative 6 months, *n* (%)				
Improvement or stabilization	69 (90.9)	35 (87.5)	34 (94.4)	0.435
Deterioration	7 (9.2)	5 (12.5)	2 (5.6)	
Surgical time, min, mean ± SD	49.9 ± 22.3	48.6 ± 22.6	51.4 ± 22.1	0.570
Postoperative hospital duration, days, mean ± SD	2.2 ± 1.6	2.3 ± 1.5	2.1 ± 1.6	0.186
Hemoglobin decline at postoperative 1 day, g/L, mean ± SD	5.8 ± 8.7	6.5 ± 9.5	4.9 ± 7.8	0.418
Success rate of UAS insertion, *n* (%)	74 (97.4)	40 (100)	34 (94.4)	0.221
Initial SFR at postoperative 1 month, *n* (%)	64 (84.2)	34 (85.0)	30 (83.3)	0.842
SFR after second procedures, *n* (%)	73 (96.1)	39 (97.5)	34 (94.4)	0.926
Postoperative main stone composition, *n* (%)				
Calcium oxalate monohydrate calculi	24 (31.6)	14 (35)	10 (27.8)	0.673
Calcium oxalate and phosphate calculi	36 (47.4)	18 (45)	18 (50)	
Uric acid calculi	6 (7.9)	2 (5)	4 (11.1)	
Mixed calculi	10 (13.1)	6 (15)	4 (11.1)	
Clavien–Dindo complications, *n* (%)				
All	12/76 (15.8)	7/40 (17.5)	5/36 (13.9)	0.666
Grade I	5	3	2	
Grade II	4	2	2	
Grade III	3	2	1	
Complications Classification				
Transient postoperative pain or fevers requiring oral analgesics or antipyretics (Grade I)	5	3	2	
SIRS/urinary sepsis requiring additional antibiotics (Grade II)	3	2	1	
Fungal infection requiring antifungal drug (Grade II)	1	0	1	
Ureteral steinstrasse requiring emergent URS (Grade III)	3	2	1	

eGFR: estimated glomerular filtration rate; BSA: body surface area; IQR: interquartile range; Δ% eGFR: (the difference between timely level and baseline level)/baseline level × 100%; UAS: ureteral access sheath; SFR: stone free rate; SIRS: Systemic Inflammatory Response Syndrome; URS: ureteroscopy lithotripsy.

### Complications

Overall, twelve patients (15.8%) developed postoperative complications, including 7 and 5 in the PS and NPS groups (17.5 vs. 13.9%, *p* = 0.666). Five cases experienced transient postoperative fevers (<38.5 °C) or pain (Grade I), and they were controlled using oral analgesics or antipyretics. Systemic inflammatory response syndrome (SIRS) or urinary sepsis (Grade II) occurred in 3 patients; they had the symptoms of obvious fever (>38.5 °C) and relevant biochemical examination indicated severe infections; the symptoms improved after using additional antibiotics like carbapenem antibiotics. One patient in the NPS group occurred postoperative fungal infection (Grade II) while this patient had a negative preoperative urine culture result. The result of fungus culture became negative when the antifungal drug (fluconazole) was given for almost 7 days. No patients need blood transfusion intraoperatively or postoperatively. Three patients (3.95%) experienced major complications with ureteral steinstrasse formation (Grade III), and emergent ureteroscopy lithotripsy (URS) was conducted to remove ureteral steinstrasse.

### Renal function changes

**Figure 1.**
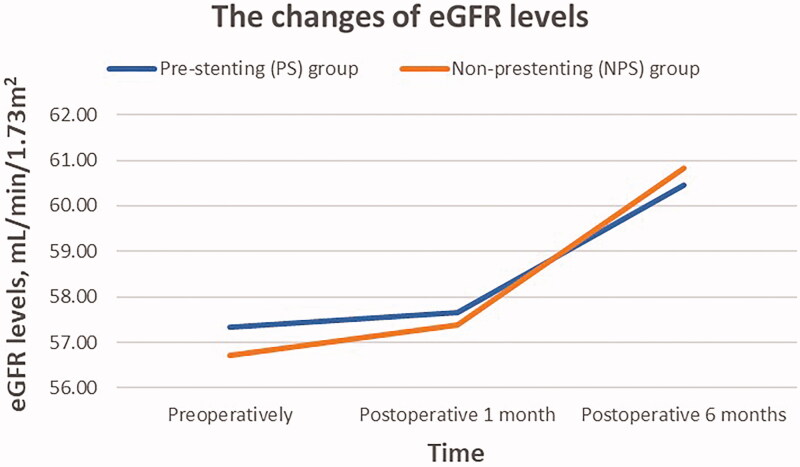
The changes in mean estimated glomerular filtration rate (eGFR) levels in the prior stenting group and non-stenting group. Both groups had an increase in mean eGFR levels compared with baseline levels.

In regards to renal function (Table 2), the mean levels of preoperative eGFR were 57.05 ± 19.37 mL/min/1.73m^2^ in total; there were 57.34 ± 19.09 and 56.73 ± 19.94 mL/min/1.73m^2^ in the PS and NPS groups, respectively (*p* = 0.891). The mean eGFR levels at postoperative 1 month were 57.52 ± 19.10 mL/min/1.73m^2^ (57.65 ±  20.27 vs. 57.38 ± 18.01, *p* = 0.952); and the mean levels at postoperative 6 months were 60.63 ± 20.54 mL/min/1.73m^2^ (60.45 ± 22.48 vs. 60.83 ± 18.46, *p* = 0.937); both were not statistically different between two groups. The changes in mean eGFR levels in the PS and NPS groups were present in Figure 1. Both groups had an increase in mean eGFR levels compared with preoperative levels. At postoperative 6 months, 87.5% of PS group patients (35/40) and 94.4% of NPS group patients (34/36) showed an improvement or stabilization in renal function, with no significant difference between the two groups (*p* = 0.435).

### The independent risk factors of renal function deterioration

Overall, seven patients developed renal function deterioration (decrease over 20% in eGFR levels) at postoperative 6 months. To identify the risk factors of postoperative renal function deterioration, all 76 patients were divided into two groups (Table 3). By using univariable logistic regression, surgical time in minutes was identified as a risk factor for deteriorated renal function (OR = 1.049, 95% CI: 1.009–1.090, *p* = 0.016, per minute), while prior stenting was not a statistically significant factor (OR = 2.429, 95% CI: 0.441–13.379, *p* = 0.308). The effect of surgical time on renal function deterioration was further analyzed after adjusting for a series of potential confounding factors. The surgical time was independently associated with the incidence of renal function deterioration by the multivariable model adjusted for preoperative greatest stone diameter, prior stenting, and preoperative eGFR levels (Table 4). The odds ratio was 1.061 (95% CI: 1.015–1.109, *p* = 0.009, per minute).

**Table 3.** The odds ratios (ORs) with 95% confidence interval (CIs) of renal function deterioration using univariable logistic regression.

Variable	Univariable logistic regression				
	Total (*n* = 76)	Deteriorated eGFR (*n* = 7)	Improved or stationary eGFR (*n* = 69)	OR (95% CI)	*p*-Value
Age, years, mean (SD)	55.0 (12.3)	52.9 (13.4)	55.3 (12.3)	0.984 (0.924–1.049)	0.622
Male, *n* (%)	43 (56.6)	4 (57.1)	39 (56.5)	1.026 (0.213–4.934)	0.975
BMI, kg/m^2^, mean (SD)	24.3 (3.7)	25.3 (3.2)	24.2 (3.8)	1.083 (0.883–1.327)	0.445
ASA grade = 2, *n* (%)	56 (73.7)	6 (85.7)	50 (72.5)	2.280 (0.257–20.208)	0.459
Hypertension, *n* (%)	21 (27.6)	1 (14.3)	20 (29.0)	0.408 (0.046–3.612)	0.421
Diabetes mellitus, *n* (%)	6 (7.9)	0	6 (8.7)	0.000 (0.000)	0.999
Positive urine culture, *n* (%)	14 (18.4)	1 (14.3)	13 (18.8)	0.718 (0.079–6.488)	0.768
Contralateral nonfunctioning kidney, *n* (%)	46 (60.5)	5 (71.4)	41 (59.4)	1.094 (0.196–6.097)	0.919
Preoperative eGFR, mL/min/1.73m^2^, mean (SD)	57.05 (19.37)	58.12 (20.41)	56.94 (19.41)	1.003 (0.963–1.045)	0.877
Greatest stone diameter, mm, mean (SD)	16.8 (4.7)	18.3 (5.8)	16.6 (4.6)	1.076 (0.919–1.260)	0.362
Patients with prior stenting, *n* (%)	40 (52.6)	5 (71.4)	35 (50.7)	2.429 (0.441–13.379)	0.308
Surgical time, min, mean (SD)	49.91 (22.27)	68.29 (10.98)	46.52 (20.00)	1.049 (1.009–1.090)	0.016

OR: odds ratios; CI: confidence interval; SD: standard deviation; BMI: body mass index; eGFR: estimated glomerular filtration rate (adjusted for body surface area); ASA Grade: Grading of patients for surgical procedures according to American Society of Anesthesiologists.

**Table 4.** Multivariable logistic regression model for renal function deterioration at postoperative 6 months.

	Multivariable logistic regression	
Variable	OR (95% CI)	*p*-Value
Greatest stone diameter, mm	1.064 (0.878–1.289)	0.525
Prior stenting, *n*	4.214 (0.522–34.027)	0.179
Preoperative eGFR levels, mL/min/1.73m^2^	1.004 (0.961–1.049)	0.842
Surgical time, min	1.061 (1.015–1.109)	0.009

OR: odds ratios; CI: confidence interval; eGFR: estimated glomerular filtration rate (adjusted for body surface area).

## Discussion

To the best of our knowledge, this is the first report on perioperative outcomes and long-term renal function changes of comparing prior stenting f-URS with non-stenting f-URS for 10–30 mm renal stones in patients with a solitary kidney. The procedure of non-stenting f-URS for renal stones in solitary kidney patients might achieve equal safety and efficacy and showed an acceptable prognosis compared with the prior stenting group in the present study. Besides, our study demonstrated that longer surgical time might be a risk factor for renal function deterioration after f-URS, while non-stenting was not a risk factor. Our findings might be significant in helping clinicians and patients with a solitary kidney make a beneficial surgical decision on the treatment of 10–30 mm renal stones.

Based on higher SFR, percutaneous nephrolithotomy (PNL) is efficient for renal stones >2 cm [12]. However, PNL in solitary kidney patients may be associated with higher bleeding risks due to higher ASA scores and compensatory dilatation of renal parenchyma [13,14]. SWL is relatively noninvasive for stones <2 cm, however, its success rate is dependent on many factors such as stone density and anatomical features [15]. Moreover, the impact on renal function after SWL is ambiguous at short-term or long-term follow-up, especially for patients with a solitary kidney [16,17]. In our department, solitary kidney patients with 1–3 cm renal stones were informed regarding available treatment options and corresponding risks before the operation. Almost all solitary kidney patients with 1–2 cm renal stones chose f-URS due to higher SFR and fewer complications. For patients with 2–3 cm renal stones, some of them chose PNL because they wanted the entire stone removed in one operation; while others requested to conduct f-URS because they could not accept the relatively greater wound and higher risks associated with PNL; moreover, they still chose f-URS even when they were informed about the possibility of multiple operations.

In the present study, overall SFR achieved 100% after an average of 1.3 procedures. This relatively high SFR could be explained as follows: (1) the mean stone size was 16.8 ± 4.7 mm, which was relatively easier to be fragmented; (2) a nitinol stone basket was used to extract stone fragments intraoperatively; (3) discharged patients were required to continue performing postural stone drainage exercises. Meanwhile, the initial and second SFRs were not statistically different between the PS and NPS groups. It seems that stenting before f-URS did not improve the SFR significantly. This result is consistent with a previous report on bilateral kidney patients [18]. However, our result was relatively significant because our study included only those patients with a solitary kidney.

The total complication rate was 15.8%, and minor complications (Grades I and II: 11.8%) were predominant. Lai et al. reported total and minor complication rates of 18.3 and 15%, respectively [3]. The complication rate was lower in our study, and that of the two groups was not statistically different. It might indicate that the PS and NPS groups presented equal safety. But three patients developed ureteral steinstrasse. They all had relatively small ureteral lumens, so stone fragments were not easily discharged. Hyams et al. showed that the incidence of ureteral steinstrasse after f-URS for 2–3 cm stones was 1.7% [19]. Therefore, to prevent postoperative ureteral steinstrasse, especially for large stones, it is better to remove the stone fragments using a stone basket as much as possible.

The total success rate of UAS insertion was relatively high (97.4%), which mainly resulted from two aspects: the use of small UAS could improve the success rate and avoid serious ureteral injury, and ureteric balloon dilation was helpful for patients with a mild and localized ureteral stricture. Most patients succeeded in inserting a UAS after attempting these methods. However, even after ureteric balloon dilation, the small UAS could not be inserted for two NPS patients due to ureteral stricture. The routine method under this circumstance was to indwell a ureteral stent and postpone the surgery. However, patients and their families were extremely worried about stone and stent-related complications. Furthermore, they would not like to perform a second operation and anesthesia due to relatively higher risks and extra costs. On their families’ request, f-URS without the UAS insertion was performed for these two patients and no serious postoperative complications occurred.

Traxer et al. reported a 46.5% ureteral injury rate when UAS was used, with the most common being perforation and stricture [20]. However, we think this rate may vary depending on surgical experience, equipment, and operating process. The methods of using small-sized UAS and ureteric balloon dilation are helpful in preventing ureteral injury. Moreover, the surgeon must be very careful in the process of pushing in and withdrawing the UAS. Violent procedures during the operation were strictly prohibited. In our study, ureteral injuries did not occur in any patients up to at least 6-month follow-up. Therefore, we consider the ureteral injury rate associated with UAS could be decreased significantly by these effective methods.

Follow-up records of the first 6 months were extracted to assess long-term renal function changes following the last procedure. Serum creatinine levels are inaccurate to assess renal function when patients experience special comorbidities such as low muscle mass and fluid overload [21]. Glomerular filtration rate (GFR) is widely regarded as a significant marker of renal function and essential for clinical research and public health [22]. Direct measurement of GFR is invasive and harmful for patients with a solitary kidney. McFadden et al. [23] conducted a systematic review and meta-analysis comparing the bias and accuracy for modification of diet in renal disease (MDRD) and chronic kidney disease epidemiology collaboration (CKD-EPI) equations; they concluded that the CKD-EPI equation could provide more accurate estimates of GFR than MDRD equation. Moreover, body surface area is another important factor in assessing GFR. Every patient’s GFR in the present study was calculated by multiplying eGFR (CKD-EPI equation) by body surface area. To a certain extent, our way of assessing GFR was more accurate than that of previous studies [3,4]. Furthermore, no statistical difference was found in renal function changes between the two groups during the 6-month follow-up; this result revealed that one-stage f-URS without prior stenting might not have a negative impact on renal function recovery postoperatively.

Operation time is an important factor affecting the outcomes and complications of f-URS [24]. According to the logistic regression analysis of our study, longer surgical time was identified as a possible risk factor for renal function deterioration after f-URS in patients with a solitary kidney. Although the mean surgical time for the PS group was relatively shorter, the median surgical time was longer (47.5 vs. 45.0 min); the difference between the two groups was relatively small and not statistically significant (*p* = 0.570). Moreover, the surgical time in the PS group did not include the duration of the pre-stenting procedure and that of removing the stent before f-URS. As a result, we could not simply conclude that the PS group had a shorter operating time than the NPS group according to the mean surgical time alone. In addition, when flexible ureteroscopy reaches the pelvicalyceal system, surgical time and SFR mainly depend on the stone characteristics and renal anatomy instead of the existence of prior stenting [25]. Therefore, it is significant to shorten the surgical time as much as possible when renal stones in patients with a solitary kidney are managed by f-URS, regardless of the existence of preoperative stenting.

The following reasons are also considered for one-stage f-URS without prior stenting. First, stenting before f-URS increases healthcare costs for the patients and workload for the clinicians; patients need pay for operation and stent-related cost; clinicians prepare and perform prior stenting procedure, resulting in less time for patients with a greater clinical need. Second, although the stenting procedure is minimally invasive and can be completed under local anesthesia, it is still an interventional operation in the urinary system; this may result in some potentially harmful stimuli and responses due to the specialty of solitary kidney patients. Third, common side effects of the indwelling ureteral stent such as irritative urinary symptoms and lumbar pain interfere with the daily activities and quality of life in up to 80% of patients [26]; serious complications like stent encrustation may result in ureteral obstruction or acute renal injury, which is relatively dangerous for patients with a solitary kidney. Last but not least, although patients without prior stenting are more likely to be detected with ureteral stenosis intraoperatively, these only accounts for a small proportion; small-sized UAS or ureteric balloon dilation is effective for most patients; therefore, prior stenting is not a prerequisite for all patients.

The historical control design of the present study might exist a risk of selection bias. However, selection bias might be relatively low since all preoperative parameters showed no statistically significant difference between the two groups. Although the same surgeon performed all surgeries and had accumulated much experience of f-URS in the early stage, we cannot ignore that the surgeon became more experienced after each operation. To a certain extent, this could potentially affect the clinical results. Finally, one-stage f-URS without prior stenting could be viable for 10–30 mm renal stones in solitary kidney patients with a good condition; however, when patients experience emergency events or severe comorbidities such as ureteral obstruction, severe infections, and acute kidney injury, prior stenting before f-URS may be relatively safe and might still be preferred by patients with a solitary kidney.

## Conclusion

Based on our single-center experience and the advantages of less surgical steps and costs, it appears that one-stage f-URS without prior stenting could be feasible for managing 10–30 mm renal stones in patients with a solitary kidney. Less surgical time might be helpful for preventing renal function deterioration after the operation. Further prospective randomized studies with more cases and multicenter are needed to verify our conclusion.

## Ethical approval

Approval was obtained from the Institutional Ethics Committee of the First Affiliated Hospital of Chongqing Medical University (Human Research Committee Approval number: 2018-003). The procedures used in this study adhere to the tenets of the Declaration of Helsinki.

## Supplementary Material

Supplemental Material

## Data Availability

The datasets used and/or analyzed during the current study are available from the corresponding author on reasonable request.

## ABBREVIATIONS

f-URS: flexible ureteroscopy lithotripsy
PS: prior stenting
NPS: not prior stenting
eGFR: estimated glomerular filtration rate
UAS: ureteral access sheath
SFR: stone-free rate
SWL: extracorporeal shock-wave lithotripsy
PNL: percutaneous nephrolithotomy
