# Supplementary material for: The feasibility of one-stage flexible ureteroscopy lithotripsy in solitary kidney patients with 1–3 cm renal stones and risk factors of renal function changes
Source: Ren Fail. 2021 Jan 24;43(1):264–72. doi: 10.1080/0886022X.2021.1872625 (PMC7850451; doi:10.1080/0886022X.2021.1872625)
Supplement: Supplemental Material [file IRNF_A_1872625_SM3156.pdf]

The equations of Chronic Kidney Disease Epidemiology Collaboration (CKD-EPI) and body surface area

Race and Sex	Serum Creatinine Level, μmol/L (mg/dL)	Equation
**Black**		
Female	≤62 (≤0.7)	$GFR = 166 \times (Scr/0.7)^{-0.329} \times (0.993)^{Age}$
	>62 (>0.7)	$GFR = 166 \times (Scr/0.7)^{-1.209} \times (0.993)^{Age}$
Male	≤80 (≤0.9)	$GFR = 163 \times (Scr/0.9)^{-0.411} \times (0.993)^{Age}$
	>80 (>0.9)	$GFR = 163 \times (Scr/0.9)^{-1.209} \times (0.993)^{Age}$
**White or other**		
Female	≤62 (≤0.7)	$GFR = 144 \times (Scr/0.7)^{-0.329} \times (0.993)^{Age}$
	>62 (>0.7)	$GFR = 144 \times (Scr/0.7)^{-1.209} \times (0.993)^{Age}$
Male	≤80 (≤0.9)	$GFR = 141 \times (Scr/0.9)^{-0.411} \times (0.993)^{Age}$
	>80 (>0.9)	$GFR = 141 \times (Scr/0.9)^{-1.209} \times (0.993)^{Age}$

$$\text{body surface area} = \text{bodyweight (kg)}^{0.425} \times \text{height (cm)}^{0.725} \times 0.007184$$
